# Global gene expression profiles from PBMCs treated with reference tobacco product preparations

**DOI:** 10.1016/j.dib.2019.103970

**Published:** 2019-05-03

**Authors:** Subhashini Arimilli, Patrudu Makena, Gang Liu, G.L. Prasad

**Affiliations:** a Eurofins Lancaster Laboratories PSS, Winston-Salem, NC 27105, USA; b RAI Services Company, 401 North Main Street, Winston-Salem, NC 27101, USA

**Keywords:** Whole smoke, Smokeless tobacco, Gene expression, PBMCs, Immune cell, Inflammatory response

## Abstract

This Data in Brief article describes global gene expression profiles from human peripheral blood mononuclear cells (PBMCs) that were treated with preparations from reference combustible and non-combustible tobacco products (TPPs). PBMCs isolated from non-smokers were treated with three non-cytotoxic doses of aqueous preparations from 3R4F cigarettes, termed Whole Smoke-Conditioned Medium (WS-CM) and a single dose of 2S3 moist snuff, termed smokeless tobacco extract (STE). PBMCs were treated with the test articles for 3 hours and the extracted total RNA was reverse transcribed and hybridized to HTA 2.0 Genechip^®^ arrays and scanned using an Affymetrix GeneChip^®^ Scanner 3000. CEL files and CHP files were generated using an Affymetrix Expression console. The CEL files were submitted to the NCBI database with GEO accession number GSE110027. The results of the microarray analyses are found in this Data in Brief article. Ingenuity Pathway Analysis (IPA; Qiagen) was used to conduct core analyses of genes that were differentially expressed by high WS-CM or STE based on the Ingenuity Gene knowledge. Expression of several of the differentially expressed genes was confirmed by RT-PCR. Analyses of these data can be found in the article “Distinct gene expression changes in human peripheral blood mononuclear cells treated with different tobacco product preparations” [1].

**Table:** Specifications table

Subject area	*Biology*
More specific subject area	*Inflammation*
Type of data	*Figures and Tables*
How data was acquired	*Gene expression profiling using Affymetrix HTA 2.0 Genechip*^*®*^*arrays and scanned using an Affymetrix GeneChip*^*®*^*Scanner 3000*
Data format	*Raw data CEL files and analyzed data are presented in excel*
Experimental factors	*PBMCs pretreated with tobacco product preparations*
Experimental features	*Gene Expression profiles from 20 samples of PBMCs treated with reference tobacco product preparations were analyzed for differential gene expression and select pathway analyses using IPA tool.*
Data source location	*NCBI database with GEO accession number**GSE110027**(*https://www.ncbi.nlm.nih.gov/geo/query/acc.cgi?acc=GSE110027)
Data accessibility	*Data is with this article*
Related research article	Arimilli S, Makena P, Liu G, Prasad GL. Distinct gene expression changes in human peripheral blood mononuclear cells treated with different tobacco product preparations. Toxicol In Vitro. *In press**[1]**.*

**Table:** 

**Value of the Data** • This Data in Brief article describes comparative gene expression differences from treatment with combustible and non-combustible tobacco products, which may aid in better understanding of the biological effects of the use of different categories of tobacco products. • The blood-derived gene expression could provide information into the systemic effects of tobacco, and can be useful in conjunction with the lung-derived data to gain more inclusive mechanistic insights into the pathophysiology leading to smoking-related diseases. • The data will be useful to achieve a better understanding of chronic inflammation.

## 1 Data

**Fig. 1:**
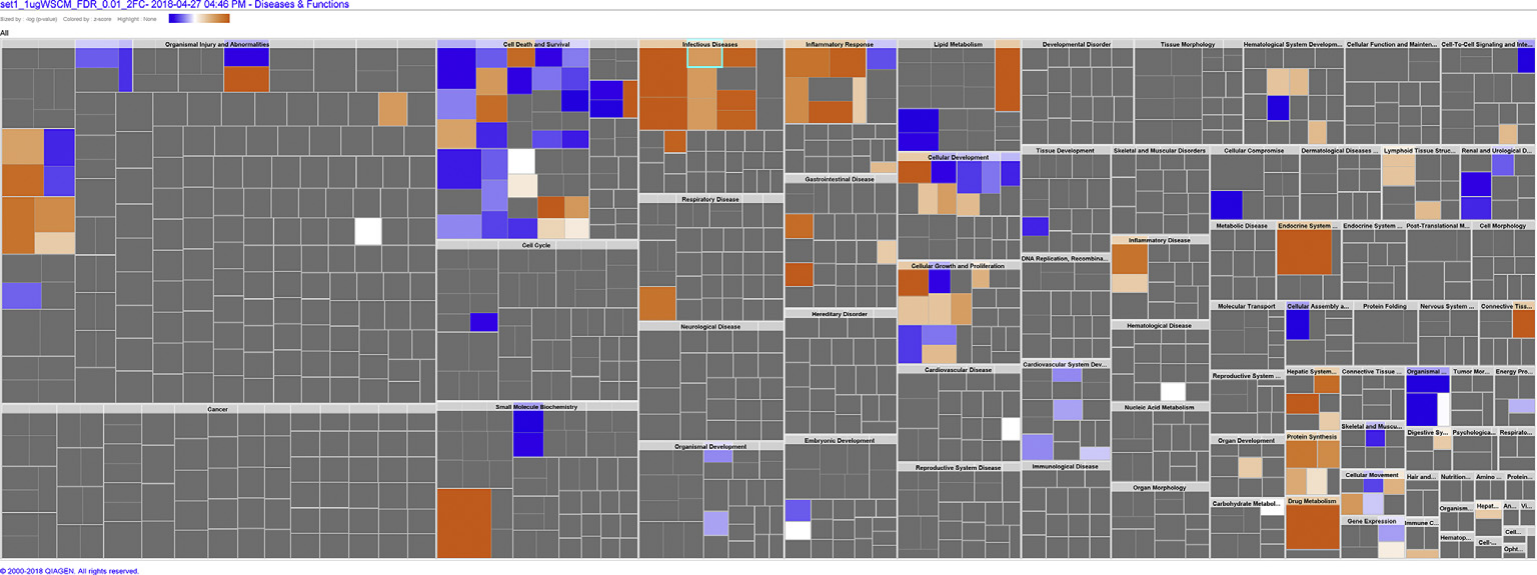
Square map showing the enriched disease and biological functions by medium WS-CM. The Square map was colored based on the z-score, which is a statistical measure of the match between expected relationship direction and observed gene expression. The color scale represents the activation z-score ranging from −1.301 (dark blue) to 1.774 (dark orange). Gray represents a z-score of 0. A z-score > 2 or < −2 is considered significant. The actual z-score is weighted by the underlying findings, the relationship bias, and dataset bias.

**Fig. 2:**
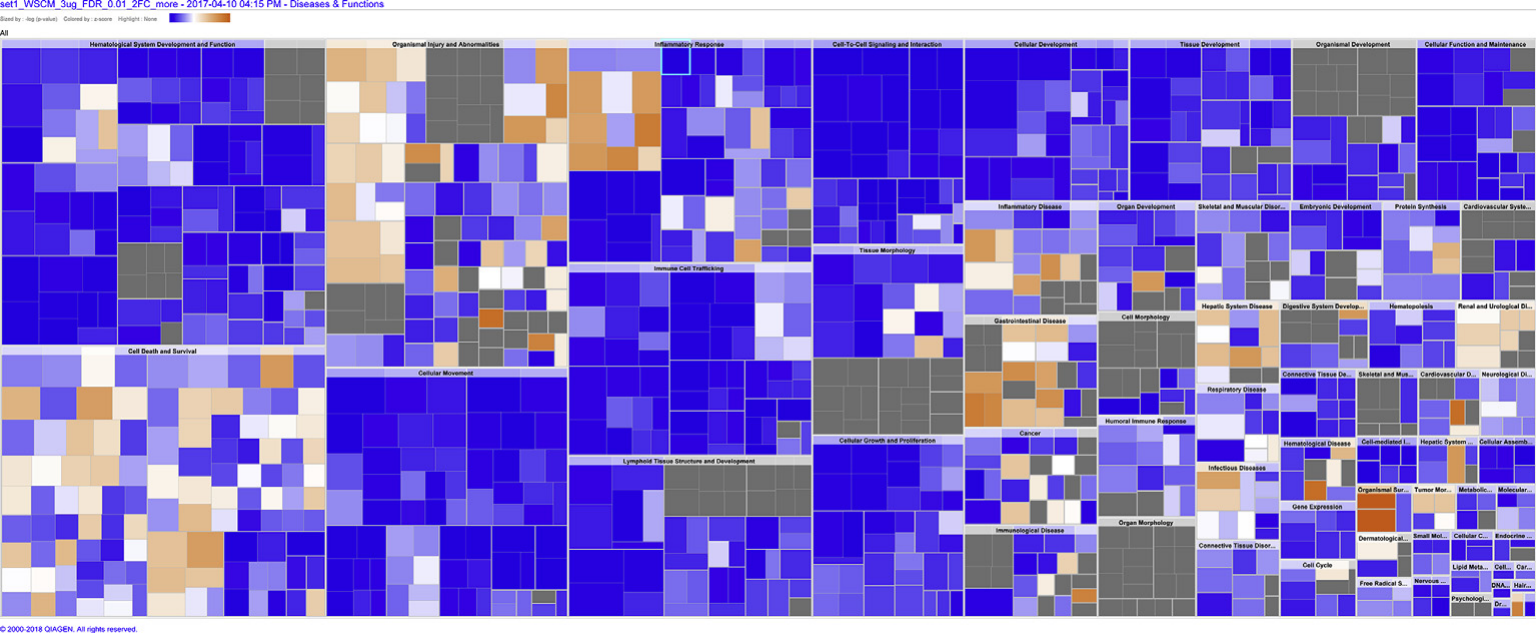
Square map showing the enriched disease and biological functions by high WS-CM. The square map was colored based on the z-score, which is a statistical measure of the match between expected relationship direction and observed gene expression. The color scale represents the activation z-score ranging from −3.244 (dark blue) to 4.902 (dark orange). Gray represents a z-score of 0. A z-score > 2 or < −2 is considered significant. The actual z-score is weighted by the underlying findings, the relationship bias, and dataset bias.

**Fig. 3:**
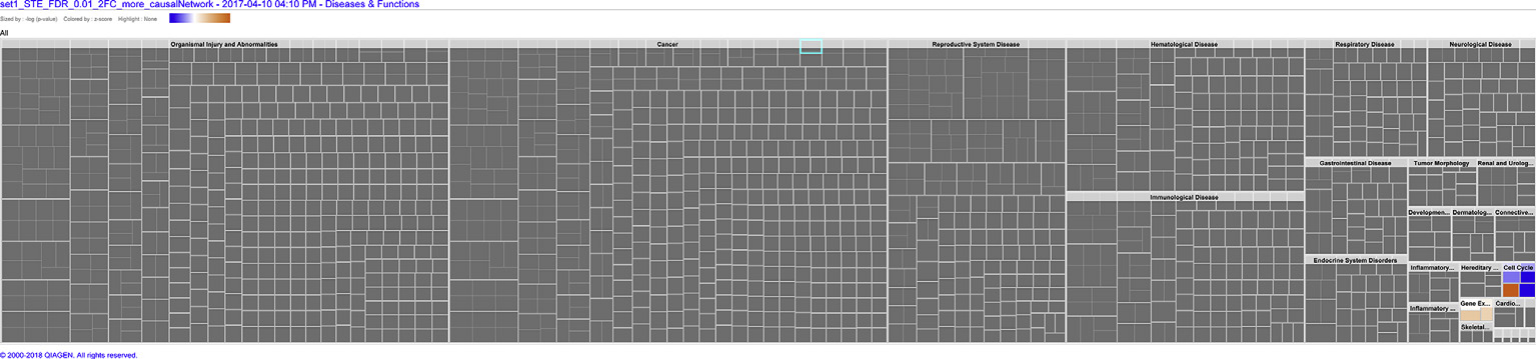
Square map showing the enriched disease and biological functions by STE. The square map was colored based on the z-score, which is a statistical measure of the match between expected relationship direction and observed gene expression. The color scale represents the activation z-score ranging from −1.785 (dark blue) to 0.212 (dark orange). A z-score > 2 or < −2 is considered significant. The actual z-score is weighted by the underlying findings, the relationship bias, and dataset bias.

The data in the tables describe 1) dose-response values for transcript up- and down-regulation, 2) pair-wise comparisons of smokeless tobacco extract (STE) and Whole Smoke-Conditioned Medium (WS-CM) treatments, and 3) transcript overlapping between medium- and high- WS-CM treatments and STE treatments. The figures illustrate the enriched disease and biological functions by medium- and high-WS-CM, as well as STE. (see Fig. 1, Fig. 2, Fig. 3)

## 2 Experimental design, materials, and methods

The experimental design is to treat peripheral blood mononuclear cells (PBMCs) with three doses (low, medium and high) of WS-CM, and at a single high dose of STE for 3 hours, which are non-cytotoxic. Isolated RNA was profiled for gene expression by microarray technology. Differentially expressed genes were identified across the three doses of WS-CM relative to untreated control conditions, and with those in STE relative to control conditions. Total number of transcripts differentially regulated by WS-CM in a dose-dependent manner (5829 transcripts upregulated and 3903 downregulated) are presented in Table S1. Pairwise comparisons of differentially regulated transcripts (>2fold) by treatments with different doses of WS-CM and STE are shown in Table S2. The overlapping transcripts that were differentially expressed between medium and high doses of WS-CM treatments with STE are summarized in Tables S3A and S3B, respectively. Additionally, pathway analyses were performed using IPA software (Qiagen).
